# Ecological resource potential

**DOI:** 10.1016/j.mex.2020.101151

**Published:** 2020-11-20

**Authors:** Harald Desing, Gregor Braun, Roland Hischier

**Affiliations:** Empa - Swiss Federal Laboratories for Material Science and Technology, Lerchenfeldstrasse 5, 9014 St. Gallen, Switzerland

**Keywords:** Earth system boundaries, Resource impacts, Precautionary principle

## Abstract

The ecological resource potential (ERP) method allows to calculate the amount of one material that can potentially be produced within Earth system boundaries, if no other anthropogenic activity would take place. It indicates the uppermost potential of one resource extracted, processed and disposed after use with a specific set of technologies and a defined probability of violating Earth system boundaries. This method is an adaption of the ecological resource availability (ERA) method, which calculates the amount of a resource that can be produced within an allocated share of the global boundaries, i.e. when considering all other anthropogenic activities. While more realistic, its allocation can be done in multiple ways and based on a variety of different objectives, which requires scenario modelling. The ERP method, in contrary, only requires information on environmental impacts from resource extraction, processing, and final disposal. The customization of the original ERA method comprises:

• Omitting all steps for allocating global boundaries to single resources or resource segments.
• Changing the calculation procedure so that ERP is calculated for each resource separately.

## Graphical abstract

**Figure:**
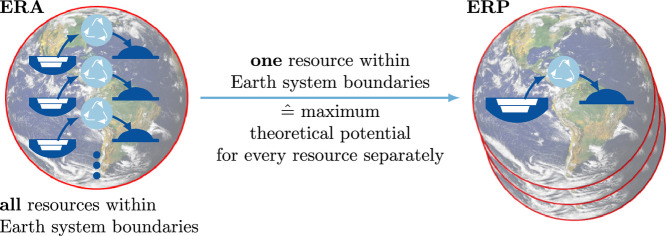

Specifications table

**Table:** 

Subject area:	Environmental science
More specific subject area:	Environmental impacts associated with extraction, processing and end-of-life treatment of primary resources
Method name:	Ecological resource potential
Name and reference of original method:	Ecological resource availability: Desing, H., Braun, G., Hischier, R., 2020. Ecological resource availability: a method to estimate resource budgets for a sustainable economy. *Global Sustainability*. https://doi.org/10.1017/sus.2020.26
Resource availability:	ERA method: https://doi.org/10.5281/zenodo.3629366 ERP method: https://doi.org/10.5281/zenodo.3827142

## Method details

The ecological resource budgets (ERB) necessary for the resource pressure design method [1] are defined either based on the ecological resource availability (ERA) or ecological resource potentials (ERP). The ERP method is a simplification of the underlying ERA method [2], therefore we provide a brief introduction and explanation of the method. For a detailed explanation, please see [2]. After the description of the ERA method, we provide details for it's adaption to calculate ERP.

### Ecological resource availability method

The extraction, processing and disposal of primary resources contributes significantly to the global environmental pressure on natural ecosystem. Reducing these burdens is a central requirement for building a sustainable circular economy [3]. And resource selection in product and service design can be one essential lever to achieve this [1]. The way we propose to aid design decision making on resource selection is to translate environmental impacts into resource budgets. To this end, we have proposed the ERA method [2]. The ERA method aims to answer the question, how much primary resources can be extracted, processed and disposed, without transgressing critical Earth system boundaries (ESB). In other words, what is a sustainable level of resource consumption by our society?

ESB represents the carrying capacity of planet Earth, i.e. the safe operating space for society [4]. Crossing these boundaries, leads to a shift of the Earth system state to a new and likely less hospitable state [5]. One proposal to quantify ESB in literature is the planetary boundaries framework [4,6]. For the purpose of the ERA method, this framework is seen as one specific set of indicators that can be extended or replaced by other boundaries. Once having described the ESB, an allocation of the boundaries to the various resource segments of the economy is necessary. This allocation step, however, is a normative choice. In other words, how much of the global safe operating space can be occupied by the extraction, processing and final disposal of a group of materials (e.g. metals)? Different allocation principles are possible, e.g. based on economic value [7] or technical feasibility [8]. In the ERA paper [2], we exemplify the allocation with a grandfathering approach, which allocates global boundaries to resource segments according to today's impact share and defining the share of production for each resource within a segment according to today's resource use pattern.

**Fig. 1:**
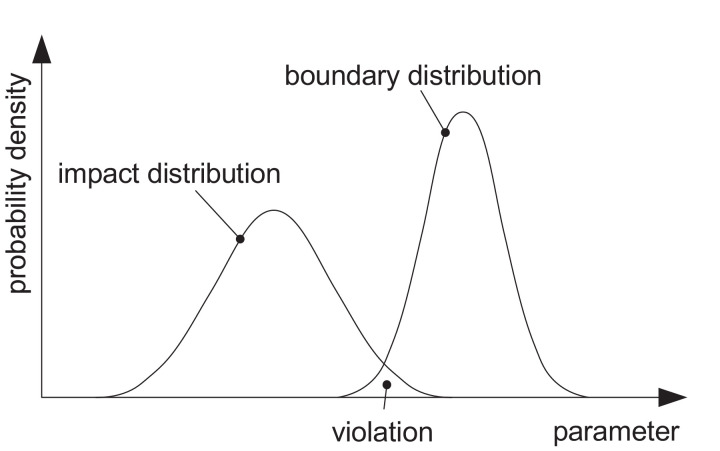
Schematic representation of the concept of ERA and ERP [2]. The resource budget for a single resource (e.g. steel) or a resource segment results from the overlap of the probability distribution of the environmental impacts with the distribution of the respective boundary. For ERA the boundary is an allocated boundary to the resource segment, for ERP it is the global boundary.

The boundaries are allocated like this to a specific resource segment and can then be used to calculate the amount of the various materials that can be produced within this segment, while staying below the boundaries. As the boundaries, their allocation and the impacts on these boundaries are uncertain; the ERA method builds on a precautionary and statistical approach. None of the boundaries is allowed to be crossed with more than a defined probability of violation (see Fig. 1). I.e. if some possible impacts are larger than possible boundary values, this boundary is violated. The probability of violation is a parameter to be chosen for the ERA method. It reflects the level of risk society is willing to accept in regard to ESB. For exemplification of the ERA method, we have chosen *P_v_* = 0*.*01 [2].

Note, all inflows into the socio-economic system will leave the system over time again as final wastes or emissions. Material cycling thereby increases the utility of material to society, however, as cycles cannot be fully closed, primary input and final disposal remain necessary parts of a CE [3,^9^,10].

In [2] the ERA method is tested for major metals used today with a grandfathering approach. The ERA budgets are listed in Table 1. They are compared to the production volume today, however, corrected with the oversize factor ω to avoid double counting. Double counting occurs when a part of a resource's production is used to produce another resource (e.g. steel necessary to produce aluminium) and impacts are accounted from cradle-to-gate [11], because they are then counted twice: once in the production of the first resource and once in the supply chain of the second. In order to avoid this problem, the oversize factor, which is the relation of how much material is produced to how much is used in sectors other than material production ($\omega ={\dot{m}}_{production}/{\dot{m}}_{output}$), allows to calculate the production output ${\dot{m}}_{output}$ to the rest of the economy [2]. The share of production indicates the relative production share of one single material to a combination of similar materials. For example, steel's share of production in the metal sector is about 90%. The resource budget for the whole resource segment *metals* is 40 times smaller than the production output in 2016. Therefore, rescaling the current socio-economic system to fit within ESB with a confidence of at least 99% requires the reduction of use of metals by 40 times. As this is not practically achievable, especially because rescaling other parts of the economy by the same factor (e.g. food) will result in a system that cannot provide for basic needs, it is necessary to optimize and refine the ERA budgets through scenarios on different allocation methods, new production technologies and optimizing the share of production.

**Table 1:** Production in 2016 ${\dot{m}}_{production,2016}$ (for cast iron [12,13] and stainless steel [14]), oversize factor *ω*[2,^11^,15], share of production (SoP) and ecological resource availability (ERA) for the investigated metals. (updated from [2]).

Metal	${\dot{m}}_{production,2016}$ (kg/a)	ω	SoP	ERA (kg/a)
Aluminium	5.89 × 10^10^	1.4	0.035	1.06 × 10^9^
Copper	2.01 × 10^10^	1.36	0.012	3.73 × 10^8^
Steel	1.61 × 10^12^	1.49	0.89	2.73 × 10^10^
Cast iron	2.48 × 10^10^	1.49	0.014	4.2 × 10^8^
Zinc	1.26 × 10^10^	1.6	0.0065	1.99 × 10^8^
Lead	4.71 × 10^9^	1.6	0.0024	7.42 × 10^7^
Tin	2.89 × 10^8^	1.6	0.00015	4.56 × 10^6^
Nickel	2.09 × 10^9^	1.91	0.0009	2.76 × 10^7^
Gold	3.11 × 10^6^	1.91	0.0000013	4.11 × 10^4^
Silver	2.57 × 10^7^	1.91	0.000011	3.39 × 10^5^
Platinum	1.91 × 10^5^	1.91	0.0000001	2.52 × 10^3^
Titanium	1.7 × 10^8^	1.91	0.00007	2.24 × 10^6^
Chromium	3.02 × 10^10^	1.91	0.013	3.99 × 10^8^
Stainless steel	4.58 × 10^10^	1.49	0.025	7.75 × 10^8^
Total for resource segment	1.81 × 10^12^	1.49	1	3.06 × 10^10^

### Ecological resource potentials

The ERP method is based on this ERA method, however, does not require any allocation. ERP describes the amount of a single material that can be extracted, produced and safely disposed within ESB, assuming that no other anthropogenic activity would exert pressure on ESB in the same time. In this way, ERP gives the maximum theoretical production potential that is possible within ESB when produced with a specified (e.g. current) technology. A material that has a large ERP has automatically a low unit impact and is therefore preferable to use in the economy. For the calculation of ERP, the following data is necessary:

• **Definition of ESB in a unit that can be measured with LCA**. Any number of boundaries in this format is acceptable to the method. The boundaries need to be specified with an uncertainty range, i.e. an interval or uncertainty distribution of likely boundary values. The following boundaries are considered in this paper, based on the planetary boundaries framework [4,6] as it is adapted in the ERA method [2]:
1. Climate change: direct fossil CO_2_ emissions to air [6,16].
2. Climate change: global warming potential for a time horizon of 100a [6,^17^,18].
3. Biosphere integrity: potentially disappeared species (reversible) [6,^19^,20].
4. Stratospheric ozone depletion: emission of O_3_ depleting substances (ODS) [6,^18^,21].
5. Biogeochemical flows: P to oceans [6,18].
6. Biogeochemical flows: P to soil [6].
7. Biogeochemical flows: industrial and intentional biological fixation of N [6,18].
8. Land system change: appropriable land area [6,^22^,23].
9. Land system change: appropriable land area for cropland [4].
10. Freshwater use: blue water consumption [6,18].
11. Energy: appropriable technical potential for renewable energy resources in electricity equivalents [22].

Any other quantitative boundary can be used in the method given that it is defined in measurable LCIA units. For example, limits to air pollution based on human health effects or local water stress can be included.

• **Unit impacts (UI) for the production of one unit of a material (usually one kilogram).** These impacts have to be reported in the same unit as the corresponding boundary and include an uncertainty distribution of the impact. UI comprise the cumulated impacts for the primary production (extraction and processing) of the material as well as for the final treatment of the same amount of material (can be incineration, landfill, wastewater treatment and/or dispersion). Data sources for unit impacts can be life cycle databases (e.g. ecoinvent [24]) or more detailed company data. The uncertainty range for each cumulative impact needs to be provided as well. For the calculations in this paper, ecoinvent v3.5 is used as a data source for unit impacts and the following LCIA and LCI results were used to compile the uncertainty range:
1. Inventory result for fossil CO_2_ emissions to air
2. GWP indicators from a variety of impact assessment methods: IPCC 2013, 2007, ILCD 2.0 2018, ReCiPe v.1.13 2016 CML 2001
3. ReCiPe v1.13 2016: endpoint ecosystem quality; uncertainty from egalitarian, hierarchic and individualist scenarios
4. Emissions of ODS expressed in CFC-11-eq; from CML 2001, ILCD 2.0 2018 and ReCiPe v1.13 2016
5. Inventory results for P and PO_4_ emissions to oceans, air, soil and freshwater. Fate factors from compartments other than ocean to ocean are taken from Doka [18].
6. Inventory results for P and PO_4_ emissions to soil.
7. Inventory results for reactive N emissions.
8. Inventory results for land occupation.
9. Inventory results for cropland occupation.
10. Inventory results for water emissions to air (evaporative water consumption) [18].
11. Cumulative energy demand in electricity equivalents, i.e. inventory results for energy carrier flows converted to electric energy [2,^22^,25].

The uncertainty of UI are built from the minimum, maximum and average (if available) of the LCIA methods considered. Additionally, the basic uncertainty of the LCI result is considered for the underlying inventory flows. The error propagation is calculated in the Monte Carlo simulation.

• **Define the probability of violation *P_v_***: The probability that impacts are higher than boundary values has to be defined. In line with the ERA method, a value of *P_v_* = 0*.*01 is chosen here.
• **Number of simulation runs *n*_runs_ for the Monte Carlo simulation**. The larger the number, the more accurate the results and the longer the simulation time. We suggest to use at least *n*_runs_ ≥ 10^5^, which leads to a simulation-to-simulation variability of the results of *<* 0*.*008 and a simulation time of *<* 30s.

For the calculation of ERP, random values are picked *n*_runs_-times for both the UI and the boundaries from among the specified uncertainty range. The initial guess for ERP (i.e. *ERP*_1_) is calculated as the fraction of the *^P^_v_/*_2_-quantile of the ESB distribution and the (1 − *^P^_v_/*_2_)-quantile of the UI distribution. The different boundary categories *k* are thereby elements of the column vector, resulting in a column vector for ERP, i.e. each element is the ERP possible within the respective boundary alone. The smallest value is the ERP for the material and the respective boundary limiting.

(1) $$ERP_{k,1}=\frac{ESB_{k}{|}_{P_{v}/2}\;}{UI_{k}{|}_{1-P_{v}/2}}$$

(2) $$ERP_{1}=\min_{k}ERP_{k,1}$$

*ERP*_1_ is then scaled up or down until the probability of violation, resulting from the overlap of UI distribution and ESB distribution, equals the required value of *P_v_* (see Fig. 1). This numerical approach is necessary, as the overlap between the two distributions depends on their shape, which is calculated numerically with the MC simulation. During this procedure the probability of violation needs to be checked for each boundary category *k*, to avoid that another boundary becomes limiting through the up or down scaling of ERP.

(3) $$ERP_{i+1}=\left\{ \begin{array}{c} ERP_{i}(1-5\;P_{v}\left(1+P_{v,i}\right)\;\;P_{v,i}>P_{v}\\ ERP_{i}\left(1+P_{v}-P_{v,i}\right)\;\;\;\;\;\;\;\;\;\;\;\;\;\;P_{v,i}<P_{v} \end{array}\right.$$

### ERP method validation

The ERP results for *P_v_* =0*.*01, calculated with process data from ecoinvent (v3.5) [24] and ESB as specified in [2], are provided for the investigated metals in Table 2. All ERP budgets for metals are limited by the CO_2_ boundary, except Cu, which is limited by the biodiversity boundary. Please note, the ERP budget for steel is smaller than global production in 2016, meaning that current production is not possible within ESB even when no other anthropogenic activity would take place.

**Table 2:** Production output in 2016 ${\dot{m}}_{output,2016}$ (i.e. production volume, see Table 1, corrected with double counting [2,^11^,15]), limiting boundary and ERP for the investigated metals (in comparison to ERA, see Table 1).

Metal	${\dot{m}}_{output,2016}$ / kg/a	limiting boundary	ERP / ^kg^/a
Aluminum	4*.*21 × 10^10^	CO_2_	4*.*36 × 10^10^
Copper	1*.*36 × 10^10^	biodiversity	5*.*68 × 10^10^
Steel	1*.*08 × 10^12^	CO_2_	4*.*99 × 10^11^
Cast iron	1*.*66 × 10^10^	CO_2_	5*.*52 × 10^11^
Zinc	7*.*88 × 10^9^	CO_2_	1*.*27 × 10^11^
Lead	2.94 × 10^9^	CO_2_	1*.*96 × 10^11^
Tin	1*.*81 × 10^8^	CO_2_	3*.*98 × 10^10^
Nickel	1*.*09 × 10^9^	CO_2_	6*.*91 × 10^10^
Gold	1*.*63 × 10^6^	CO_2_	5*.*92 × 10^7^
Silver	1*.*35 × 10^7^	CO_2_	2*.*85 × 10^9^
Platinum	1.00 × 10^5^	CO_2_	3*.*26 × 10^7^
Titanium	8*.*9 × 10^7^	CO_2_	2*.*88 × 10^10^
Chromium	1*.*58 × 10^10^	CO_2_	3*.*14 × 10^10^
Stainless steel	3.07 × 10^10^	CO_2_	1*.*91 × 10^11^

**Fig. 2:**
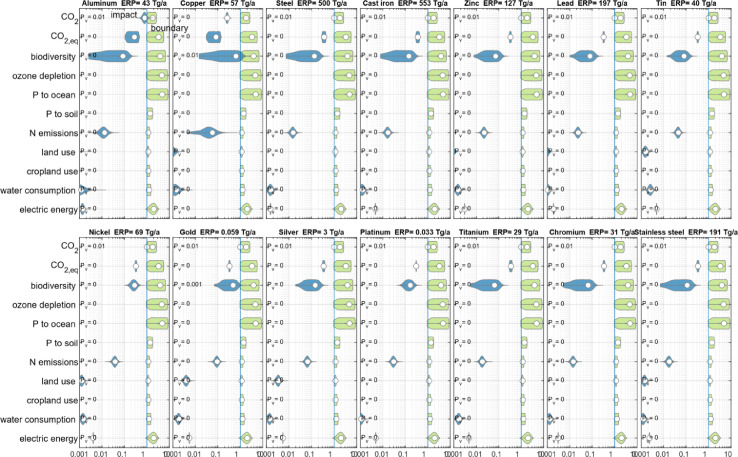
Graphical representation of probability density function (PDF) of impacts (blue) on global boundaries (green) for each boundary category and each ERP for selected metals.

The results for each metal can also be displayed with the overlapping probability density functions of impacts and boundaries (see Fig. 2). One boundary category is always limiting, when the probability density functions of impacts and boundaries overlap with a probability of *P_v_* = 0*.*01. In some cases, also a second boundary (or possibly more) overlap with a probability of *P_v_ <* 0*.*01 (see e.g. case of Gold). Generally, for most boundaries the PDFs do not overlap, i.e. *P_v_* = 0.

## Conclusions

The here presented ERP method allows to estimate how much of a resource could be theoretically produced without violating any of the specified Earth system boundaries. Due to the focus on a single material, this value does not suggest a practical resource budget, but rather reflects the impact intensity of that material. The advantage of the method over a simple comparison of impact intensities is that different impact categories can be considered and automatically the most pressing environmental concern is limiting ERP. Different resources can then be compared to each other independent on the limiting boundary, as the resource potentials are all in the same units.

## Declaration of Competing Interest

The authors declare that they have no known competing financial interests or personal relationships that could have appeared to influence the work reported in this paper.
